# Novel Discoveries in Immune Dysregulation in Inborn Errors of Immunity

**DOI:** 10.3389/fimmu.2021.725587

**Published:** 2021-08-27

**Authors:** Anwen Ren, Wei Yin, Heather Miller, Lisa S. Westerberg, Fabio Candotti, Chan-Sik Park, Pamela Lee, Quan Gong, Yan Chen, Chaohong Liu

**Affiliations:** ^1^Department of Pathogen Biology, School of Basic Medicine, Tongji Medical College, Huazhong University of Science and Technology, Wuhan, China; ^2^Wuhan Children’s Hospital, Tongji Medical College, Huazhong University of Science and Technology, Wuhan, China; ^3^The Laboratory of Intracellular Parasites, Rocky Mountain Laboratories, National Institute of Allergy and Infectious Diseases, National Institutes of Health, Hamilton, MT, United States; ^4^Department of Microbiology Tumor and Cell Biology, Karolinska Institutet, Stockholm, Sweden; ^5^Division of Immunology and Allergy, Lausanne University Hospital and University of Lausanne, Lausanne, Switzerland; ^6^Department of Pathology, Asan Medical Center, University of Ulsan College of Medicine, Seoul, South Korea; ^7^Department of Paediatrics and Adolescent Medicine, Li Ka Shing Faculty of Medicine, The University of Hong Kong, Hong Kong, China; ^8^Department of Immunology, School of Medicine, Yangtze University, Jingzhou, China; ^9^Clinical Molecular Immunology Center, School of Medicine, Yangtze University, Jingzhou, China; ^10^The Second Department of Pediatrics, Affiliated Hospital of Zunyi Medical University, Zunyi, China

**Keywords:** inborn errors of immunity, immune dysregulation, primary immune dysregulation disease, autoimmunity, hyperinflammation, lymphoproliferation

## Abstract

With the expansion of our knowledge on inborn errors of immunity (IEI), it gradually becomes clear that immune dysregulation plays an important part. In some cases, autoimmunity, hyperinflammation and lymphoproliferation are far more serious than infections. Thus, immune dysregulation has become significant in disease monitoring and treatment. In recent years, the wide application of whole-exome sequencing/whole-genome sequencing has tremendously promoted the discovery and further studies of new IEI. The number of discovered IEI is growing rapidly, followed by numerous studies of their pathogenesis and therapy. In this review, we focus on novel discovered primary immune dysregulation diseases, including deficiency of SLC7A7, CD122, DEF6, FERMT1, TGFB1, RIPK1, CD137, TET2 and SOCS1. We discuss their genetic mutation, symptoms and current therapeutic methods, and point out the gaps in this field.

## Introduction

The immune system is under regulation of several checkpoints during central and peripheral development. Disorder of regulation can cause abnormal activation and expansion of immune cells, leading to autoimmunity, hyperinflammation and even malignant proliferation. Inborn errors of immunity (IEI), used to widely known as primary immunodeficiency (PID), was historically defined by higher susceptibility to infections due to monogenic germline mutations. However, recent studies reveal that immune dysregulation accounts for a large proportion of manifestations in PID patients (1, 2). Additionally, it results in a worse prognosis in patients with immune dysregulation compared to those with only high infection susceptibility (3). International Union of Immunological Societies (IUIS) lists “Diseases of immune dysregulation” as an independent category of IEI (1), including familial hemophagocytic lymphohistiocytosis (FHL syndromes), FHL syndromes with hypopigmentation, regulatory T cell defects, autoimmunity with or without lymphoproliferation, immune dysregulation with colitis, autoimmune lymphoproliferative syndrome (ALPS, Canale-Smith syndrome) and susceptibility to EBV and lymphoproliferative conditions. Patients with these diseases suffer a combined manifestation of immune deficiency, autoimmunity, recurrent inflammation, lymphoproliferation and even predisposition to malignancy. Their immune cells like B cell, T cell and NK cell have abnormal amounts and functions. Due to the coexistence of autoimmunity and immunodeficiency in some cases, clinical treatment requires a delicate balance. Hematopoietic stem cell transplantation (HSCT) is a potential therapy, but improving survival rate still remains an issue (4). Considering its poor prognosis and difficult treatment, a deeper understanding of immune dysregulation in IEI is required for precise and timely diagnosis, disease monitoring and therapy.

Since the number of cases for any particular disease is usually few, a large-scale study of IEI can hardly be carried out. Thus, there is difficulty in studying and curing these diseases. With technical advancements in whole-exome sequencing/whole-genome sequencing (WES/WGS), tremendous progress has been made in the identification of mutations causing IEI, whose number has doubled in ten years (from 2009 to 2019) and continues to increase rapidly (1, 2). Here, we review novel discoveries of immune dysregulation in IEI, based on the genes listed in the Table 4 of IUIS 2019 IEI report (1) and its 2020 interim update (2), including SLC7A7 deficiency (FHL syndromes), CD122 deficiency (regulatory T cell defects), DEF6 (regulatory T cell defects), FERMT1 (regulatory T cell defects), SOCS1 (autoimmunity with or without lymphoproliferation), TGFB1 (immune dysregulation with colitis), RIPK1 (immune dysregulation with colitis), CD137 (susceptibility to EBV and lymphoproliferative conditions) and TET2 (susceptibility to EBV and lymphoproliferative conditions) (**Table 1**). We make a thorough review about their genetic mutations, clinical phenotypes and possible treatments.

**Table 1:** Newly reported primary immune dysregulation diseases.

Disease	Gene defect	Number of patients/families	Consanguinity (numbers of consanguineous families/numbers of non-consanguineous families)	Mode of inheritance	Molecular manifestation	Cellular manifestation	Clinical symptoms						Immune labs	Treatment			Outcomes (alive/dead)	Reference
							Immunodeficiency	Autoimmunity	Inflammation	Malignancy	Lymphoproliferation	Others		HSCT	Target treatment	Others		
SLC7A7 Deficiency	*SLC7A7*	16/9, 28/NR	6/3	AR	Deficiency in y^+^LAT-1 causes manifested amino acid transport	Impaired absorption of CAAs in intestinal epithelium cells, impaired resorption in renal tubular epithelium cells, increased cell apoptosis of epithelial cells, impaired macrophage phagocytosis, aberrant TLRs pathways	Yes, Organ: pulmonary diseases	Yes, especially lupus nephritis, vitiligo and immune thrombocytopenic purpura	Yes, HLH	NR	Yes, HM, SM	LPI, cardiovascular diseases	Cationic amino-acids levels ↓; BS ↑, D-dimer↑, PAP↑, F1+2 ↑; Leukocyte ↓, thrombocyte ↓, anemia	NR	Anti-cytokines drugs	Prevention of hyperammonemia; nutritional supplementation; prevention of specific complications, such as renal and cardiovascular manifestations	38/6	(5–7)
CD122 Deficiency	*IL2RB*	10/5	5/0	AR	Dysfunctional IL-2R causes dysregulated IL-2/15 signaling, elevated plasma IL-2/15 levels	Dysregulated innate and adaptive immune function	Yes, Organ: pneumonitis, otitis media, urinary tract infection, gastroenteritis and dermatitis Infectious microbes: EBV, CMV	Yes, AIHA, autoimmune enteropathy	Yes, Early onset IBD	NR	Yes, LAD, HM, SM, large tonsils	Food allergy, eczema, failure to thrive	CD8^+^ T cells ↑, memory T cells ↑, Tregs ↓, NK cells ↑, Ig ↑, Coombs test +	Yes, 2 alive and 2 dead	Hyper-stimulate residual surface IL-2Rβ using IL-2 anti–IL-2 antibody complexes, IL-2 superkine, orthoIL-2 analogues or IL-2 Fc fusion proteins	Methylprednisolone for management of autoimmunity	3/7	(8, 9)
DEF6 Deficiency	*DEF-6*	7/3	3/0	AR	Disruption in CTLA-4 traffic	Over-activation of T cells	Yes, Organ: sepsis, respiratory Infectious microbe: CMV, EBV, respiratory syncytial virus, rotavirus, rhinovirus, influenza B; S. pneumoniae, S. aureus, S. epidermis, E. aerogenes, E. cloacae, E. faecalis, E. aerogenes, K. oxytoca, S. epidermis, E. faecalis	Yes, AIHA	Yes, recurrent fevers, IBD	Yes, EBV+ nodular sclerosis classic Hodgkin lymphoma	Yes, HM, SM	Dilated cardiomyopathy in some patients	CD4^+^ T cells ↓, thrombocytes ↓, ANCA and autoantibodies +	Yes, Auto-HSCT 1alive	CTLA-4-Ig therapy,	Immunosuppressants, plasma exchanges	6/1	(10, 11)
FERMT1 Deficiency	*FERMT1*	19/10	2/1, 7 NR	AR	Reduced integrin activation, higher ROS concentration	Reduced keratinocyte- ECM adhesion, reduced epidermal keratinocytes proliferation, fibroblasts stimulated.	NR	NR	Yes, colonic inflammation, gingivitis, periodontitis and mucosal inflammation	Higher risk of SCC	NR	Skin atrophy, blistering, poikiloderma, photosensitivity, mucosal stenosis	Immunofluorescence shows structure abnormality	NR	NR	Gene therapy and protein replacement may be useful	19/2	(12–14)
SOCS1 Deficiency	*SOCS1*	12/7	NR	AD	Enhanced STAT1 phosphorylation and a proapoptotic transcriptional signature	Increased sensitivity to interferons	Only two of the patients are reported to have severe infection history, one with COVID-19 and one with bronchopulmonary	Yes, AIHA, ITP, polyarthritis, psoriasis	Yes, multisystem inflammation like fever	NR	Yes, lymphoproliferation	NR	Neutrophils ↓, lymphocytes ↓, Ig ↓, autoantibodies +,	NR	SOCS1 mimetic peptide and JAK1/2 inhibitor baricitinib	Corticosteroids, mycophenolate mofetil	12/0	(15, 16)
TGFB1 Deficiency	*TGFB1*	3/2	1/1	AR	Defective TGFB1 signaling and reduced phosphorylation of SMAD2/3 in lamina propria mononuclear CD45^+^ CD19^+^ and CD3^+^ cells	T cells fail to activate and proliferate properly after anti-CD3/anti-CD28 or specific antigens stimulation	Yes, Organ: LRTI, URTI, retinitis Infectious microbe: CMV	NR	Yes, infantile IBD	NR	NR	CNS disease associated with epilepsy, brain atrophy and posterior leukoencephalopathy	T cell proliferation under stimulation of CD3 ↓	NR	Recombinant TGF-β1 replacement	Surgery, nutritional therapy and fecal microbiota transplantation targeting early onset IBD; anti-inflammatory therapy	1/2	(17)
RIPK1 Deficiency	*RIPK1*	13/10	3/1, 6 NR	AR	Impaired proinflammatory signaling	Dysregulated cytokine releases such as increased IL-1β and decreased IL-10, higher levels of inflammasome activity upon stimulation, enhanced necroptosis	Yes, Organ: thrush mycotic stomatitis, enteritis, pneumonia, conjunctivitis. Infectious microbe: candida albicans,	NR	Yes, recurrent fever, early-onset IBD	NR	Yes, HM, SM	NR	Naïve CD4^+^ T and naïve CD8^+^ T, B and NK cells ↓, IL-1β ↑	Controversial, 1 alive	NR	Surgery for IBD, improvement pulmonary hypertension, drugs protecting liver, intravenous injection of globulin and antibiotics to resist the bacterial infection	7/6	(18–20)
CD137 Deficiency	*TNFRSF9*	6/6	5/1	AR	Impaired cositmulation, mitochondrial respiration, biogenesis and function	Impaired T-cell survival, proliferation, and cytotoxicity; Diminished NK cell function; Reduced B cell activation, proliferation, and class switch recombination	Yes, Organ: sinopulmonary infections, bronchiectasis, Infectious microbes: EBV	Yes, AIHA	Yes, persistent fevers, HLH	Yes, high predisposition to EBV-related B-cell lymphoma	Yes, HM, SM, LAD	NR	Proportions of transitional and immature B ↑, memory B cells and plasmablasts ↓, NK cells ↓, TFHs ↓, proportion of Tregs ↓, Ig ↓,	Yes, 1 alive	Other costimulators like CD28	Immunosuppression, antibiotic prophylaxis, regular immunoglobulin substitution, anti-CD20 mAb (rituximab) and chemotherapy	6/0	(21–23)
TET2 Deficiency	*TET2*	6/3	2/0, 1 NR	AR	Increased level of DNA methylation causes failure of transcription factors binding	Impediment in GC exit, antigen presentation and differentiation of GCB cells	Yes, Organ: LRTI Infectious microbe: RSV, CMV, EBV	Yes, autoimmune cytopenias	Yes, HLH	Yes, remarkable predisposition to lymphoma	Yes, LAD, HM, SM	Growth impairment, variable thyroid diseases	DNT ↑, class-switch memory B cell ↓, sFasL ↑,	Yes, 2 alive, 2 dead	Inflammatory signals inhibition	Vitamin C treatment, infection prevention; Intravenous immunoglobulin, rituximab (anti-CD20 antibody) and corticosteroid	4/2	(24, 25)

AD, autosomal dominant; AIHA, autoimmune hemolytic anemia; ANCA, anti-neutrophil cytoplasmic antibodies; AR, autosomal recessive; BS, bleeding score; CMV, cytomegalovirus; EBV, Epstein-Barr virus; HLH, hemophagocytic lymphohistiocytosis; HM, hepatomegaly; IBD, Inflammatory bowel disease; LAD, lymphadenopathy; LPI, lysinuric protein intolerance associates; LRTI, lower respiratory tract infection; NR, none reported; F1+2, circulating prothrombin fragment 1 and 2; PAP, plasmin-α2-antiplasmin; SCC, squamous cell carcinoma; SM, splenomegaly; URTI, upper respiratory tract infection.

## SLC7A7 Deficiency

Solute carrier family 7A member 7 *(SLC7A7)* encodes y^+^L amino acid transporter-1 (y^+^LAT-1). It is expressed mainly in monocyte‐derived macrophages, as well as intestinal and renal cells, while its homolog y^+^LAT-2, encoded by *SLC7A6*, is expressed ubiquitously, but with low levels in the cells mentioned above, explaining its inability to compensate for y^+^LAT-1 in LPI (26).

*SLC7A7* mutations contribute to lysinuric protein intolerance (LPI), whose symptoms compose of growth retardation, muscle hypotonia and hepatosplenomegaly (27). More related manifestations were revealed in later studies, such as pulmonary diseases, cardiovascular diseases, hemophagocytic-lymphohistiocytosis (HLH) and autoimmune diseases, especially lupus nephritis, vitiligo and immune thrombocytopenic purpura (5). It was initially thought that y^+^LAT-1 deficiency in polarized cells such as intestinal and renal tubular epithelium cells is the pathogenesis of LPI, since impaired absorption of cationic amino acids (CAAs) in the intestinal epithelium and impaired resorption in the kidneys cause an imbalance of amino acids, reduction of protein synthesis, hyperammonemia and growth disorders. However, later studies revealed that non-polarized cells like lymphocytes and macrophages are also involved, which contributes to renal, pulmonary, and immune disorders (28, 29). Immune dysregulation in LPI patients appears as a decrease in leukocyte phagocytic, cytotoxic, and natural killer (NK) cell activity and an increase in spontaneous proliferation of lymphocytes (28). Impairment of macrophage phagocytosis by amino acid transport leads to not only higher susceptibility to viral infection, but also abnormal inflammatory state and autoimmune diseases, since aberrant phagocytosis fails to remove apoptotic cells, which are related to inflammation and autoimmune responses (29). Furthermore, intracellular arginine of epithelial cells is accumulated since the influx of CAAs in LPI cells is intact, while efflux is abolished, possibly producing more nitric oxide (NO), which induces cell damage and apoptosis. Finally, cell responses to inflammatory and apoptosis increase (6). Together, increases in cell apoptosis and decreases in its clearance result in aberrant inflammation and autoimmunity. Apart from reduced phagocytosis, studies also demonstrate that there are other macrophage dysfunctions, such as aberrant toll-like receptor (TLR) pathways and a rise in serum inflammatory cytokine levels (30). These findings elucidate a central role of macrophages and the innate immune system in the pathogenesis of LPI. Recent studies reveal more functions other than arginine transposition of y^+^LAT-1. For example, the *SLC7A7* mutation directly provokes production of proinflammatory cytokines, IL-1β and TNF-α, in macrophages and airway epithelial cells in an arginine independent way, partially explaining HLH and pulmonary diseases in y^+^LAT-1 deficient patients. This may ascribe to y^+^LAT-1’s ability to inhibit the nuclear factor kappa-B (NF-κB) pathway in a physiological scenario (31). Once this inhibition is lifted, cytokines are produced and released in large amounts. Besides, bleeding events were reported in some cases (7). Patients do not have spontaneous bleeding tendency but mucocutaneous bleeds can be triggered by invasive and surgical interventions or postpartum. Possible mechanisms include reduced NO production (32) and impaired hepatic clearance of F1 + 2, PAP, and D-dimer (7).

Difficulty in diagnosis lies in the heterogenetic phenotypes. Patients with the same point mutation (6) or even from the same family (5)may have different clinical manifestations and prognosis. Therefore, the *SLC7A7* mutation is the only precise diagnostic method, but it still cannot predict the symptoms and disease development (5). Current therapy consists of three parts: ① prevention of hyperammonemia, meaning that hypoproteinemic regimen is required; ② nutritional supplementation, including L-citrulline, L-carnitine, vitamins and other nutritional supplementation; ③ prevention of specific complications, like renal and cardiovascular manifestations (5). With the development of our knowledge of LPI, new therapeutic strategies, like anti-cytokine drugs are proposed as well (31). Notably, pulmonary involvement is highly associated with death (5) and therapy targeting pulmonary is necessary.

Even though LPI was discovered more than fifty years ago, its pathogenesis still remains unclear. The relationship between *SLC7A7* and LPI requires further study. The tamoxifen-induced ablation by UBC-Cre-ERT2 of Slc7a7 in the mouse (Slc7a7^−/−^) model reported by Bodoy et al. successfully mimics the phenotypes in human LPI (33). Viable animal models like this may accelerate the research of LPI pathogenesis, especially the complicated immune manifestations and possible available treatment.

## IL-2Rβ (CD122) Deficiency

IL-2 is a critical immune regulation cytokine and IL-2Rs are expressed on the surface of T cells and NK cells, which are composed of IL-2Rα (CD25), IL-2Rβ (CD122) and IL-2Rγ (CD132). IL-2 is bi-functional in immune regulation. First of all, it boosts the immune response by promoting the proliferation, differentiation and function of effector T cells and NK cells (34, 35). Secondly, it participates in the maintenance and function of Tregs (regulatory T cells) which act as suppressive regulators (35, 36). In recent years, IL-2 and related biological production began to be used in the treatment of cancer and autoimmune diseases (37, 38). For the best application of these drugs, a full understanding of how IL-2 functions through IL-2Rs is imperative and the study of IL-2R deficiency can improve it vastly.

While the roles of IL-2Rα and IL-2Rγ deficiency in IEI have been known for a long time, IL-2Rβ deficiency in humans was just reported recently. Apart from IL-2R, IL-2Rβ also participates in the formation of IL-15R. Early studies revealed a connection between IL-2Rβ and autoimmune diseases like rheumatoid arthritis (RA) and type 1 diabetes (TID) (39, 40). Additionally, studies on IL-2Rβ deficient patients further confirm the connection and enrich the spectrum of primary diseases of immune regulation. Fernandez et al. reported a pair of siblings with homozygous *IL2RB* mutations which decrease IL-2Rβ expression and dysregulate IL-2 and IL-15 signaling. Plasma levels of IL-2 and IL-15 are increased, thus explaining the elevated CD8^+^ T cells and NK cells, while other proinflammatory cytokines are almost equal to healthy controls. Although the total number of NK cells in the patients is elevated, there is a block in the transition from immature ones to functional ones, contributing to higher susceptibility to cytomegalovirus (CMV). As for T cell subsets, there is a skew to memory T cells with reduced Tregs (8). Another group (9) reported patients with different mutations in *IL2RB*. Despite a slight difference in phenotypes and severity, all patients share common manifestations, namely immunodeficiency and autoimmune diseases including enteropathy, skin abnormalities, autoimmune hemolytic anemia, hypergammaglobulinemia and high susceptibility to infections, proving the pleiotropic functions of IL-2R signaling.

HSCT has a therapeutic effect on IL-2Rβ deficiency, but also causes a risk of complications of thrombotic microangiopathy, which can be lethal (8, 9). Another potential treatment is to hyper-stimulate residual surface IL-2Rβ, since expression of IL-2Rβ is decreased rather than abolished and downstream signaling pathways remain intact (9).

## DEF6 Deficiency

Differentially expressed in FDCP6 homolog (DEF6), also known as IRF4 binding protein (IBP) or SWAP-70-like adaptor of T cells (SLAT), is a TCR downstream guanine nucleotide exchange factor (GEF).

Variations in phenotypes of *DEF-6* knockout mice made its immune function mysterious (41–43). However, studying inborn DEF6 deficient patients clarified its role. Clinical manifestations of DEF6 deficient patients consist of T-cell lymphopenia, low class-switched B cells, hepatosplenomegaly, autoimmunity, bowel inflammation and susceptibility to EBV (10, 11). Cytotoxic T Lymphocyte antigen 4 (CTLA-4), expressed by activated T cells and Foxp3^+^ Tregs, is an antagonist of co-stimulator CD28 and competes with it in combination with CD80/CD86. Therefore, it has a negative role in co-stimulation of T cells (44). Membrane expressed CTLA-4 undergoes endocytosis constitutively and therefore CTLA-4 in healthy people is dominantly located in intracellular vesicles. Internalized CTLA-4 either goes back to the plasma membrane or is degraded (44). Previous studies have already shown that deficiency in lipopolysaccharide-responsive and beige-like anchor protein (LRBA), a protein involved in CTLA-4 traffic, causes increased CTLA-4 degradation and patients with LRBA deficiency have autoimmune and inflammatory symptoms just like those with DEF6 deficiency (45). Patients with DEF6 deficiency also show reduced availability of surface CTLA-4. DEF6 directly interacts with the small GTPase, RAB11, on recycling endosomes and therefore affects CTLA-4 shuttling. The attenuated availability of CTLA-4 accounts for defected CD80 uptake and autoimmune symptoms of the patients (10). In addition, it may also thwart Tregs maturation because Foxp3^+^CD25^-^ regulatory T cells, likely being immature Tregs or precursors, are over-expanded in DEF6 deficient patients (11).

CTLA-4-Ig therapy helps to ameliorate these symptoms (10) and plasma exchanges and immune suppressors like corticosteroids, rituximab, azathioprine and bortezomib are also shown to be effective (11).

## FERMT1 Deficiency

Mutations in FERMT1 (also known as KIND1), encoding the focal adhesion protein kindlin-1, cause Kindler syndrome (KS). KS patients are predominantly offspring of consanguineous couples (12) but there are exceptions (46). The major manifestation of KS is skin disorder such as atrophy, blistering, poikiloderma, photosensitivity (12, 13, 47). There is also increased risk of mucosal stenosis and muco-cutaneous cancer in KS patients. Extra-cutaneous manifestations mainly lie in inflammation, including colonic inflammation, gingivitis, periodontitis and mucosal inflammation (13). FERMT1 is critical to integrin activation. When FERMT1 is deficient, reduced β1 integrin activation causes attenuated keratinocyte-cell-extracellular matrix (ECM) adhesion, partially explaining the skin blistering in KS (47). Also, reduced β1 integrin activation is related to lower epidermal keratinocytes proliferation, accounting for skin atrophy in KS (47). While atrophy and blistering often occur at young patients, photosensitivity and squamous cell carcinoma (SCC) happen later. This is because with the patients aging, the effect of UV irradiation and chemical stressors accumulates, provoking reactive oxygen species (ROS). ROS leads to oxidative stress and molecular damage including DNA damage. Normal people can be protected from these damages by FERMT1 through activation of ERK pathway and inhibition of cyclin‐dependent kinase (CDK) activity while in KS patients, they accumulate and result in photosensitivity and high risk of SCC (48–50). Others hold the view that FERMT1 exerts a tumor-suppression role by balancing TGF-β–mediated growth-inhibitory signals and Wnt–β-catenin–mediated growth-promoting signals. Loss of balance when FERMT1 is deficient results in higher risk of muco-cutaneous cancer (51). However, in this animal experiment, tumors induced are basal cell carcinomas but not SCC. Inflammation of KS consists of increased cytokine secretion and macrophage infiltration but the precise mechanisms have not been revealed (52). Mucosal stenosis indicates the existence of fibrosis. Further studies reveal paracrine epithelial–mesenchymal signals accounting for the fibrosis. Keratinocytes lack FERMT1 over express and secret IL-20 and IL-24 under pressure, which stimulate fibroblasts and promote fibrosis (52). Genotype–phenotype correlation is not clear in KS, and possible influential factors consist of environmental, ethnic and geographical backgrounds (14). Studies on immune system in KS patients are rare up to now. However, integrin β1 is critical for CD4^+^ T cells migration (53), indicating that this process is possibly affected in KS patients.

Like many other IEI, mutation analysis is the most reliable diagnosis method for FERMT1 deficiency. Disrupted basement membrane found by indirect immunofluorescence (IIF) and transmission electron microscopy (TEM) also provides supportive evidence (54). It is same to other epidermolysis bullosa(EB) that there has not been a widely accepted, specific, safe and effective therapeutic method for KS, although gene therapy, protein replacement and HSCT have been reported (55). Further studies focusing on the exact pathogenesis of KS and precise functions of FERMT1 will shed light on this field.

## SOCS1 Deficiency

Interferons (IFN) are critical for activating immune responses and providing antiviral protection. By binding to the receptor complex, they activate JAK, which then phosphorylates STAT. Activated STAT translocates to the nucleus and changes the expression of genes, contributing to phenotype alterations. However, their roles are not always beneficial to the host. Excessive IFN signaling causes severe toxicity and even lethal syndromes (56, 57). Consequently, a balance between activation and suppression of IFN is required. Suppressor of cytokine signaling (SOCS) 1 is an essential suppressor for type I and type II IFN signaling through inhibiting the JAK-STAT pathway and its expression can be induced by cytokines including type I and type II IFN, forming a negative feedback loop (58, 59). Homozygous deficiency of SOCS1 in mice is lethal with hypersensitivity to IFN-γ (57) and there are no homozygous SOCS1 deficient patients reported up to now, indicating its indispensable and uncompensated role in immune homeostasis. Mechanisms of SOCS1 in inhibiting type I and type II IFN signaling are quite different. As for type II IFN, both expression and sensitivity are increased in SOCS1 deficient mice, while only sensitivity is affected in the case of type I IFN (60). Among 8 family members of SOCS proteins, SOCS1 and SOCS3 are distinctive in inhibitory mechanisms. They not only inhibit downstream signaling by facilitating ubiquitination of signal intermediates, but they also have a kinase inhibitory region (KIR) that interacts with JAK directly. SOCS1 acts as a pseudo-substrate of JAK, blocking its interaction with real substrates, which mediate downstream signals. SOCS1 also binds to unphosphorylated JAK, which enhances its negative regulatory role (61). Also, accumulating studies show that SOCS1 can function in the nucleus in a different mode with JAK-STAT inhibition. It has a nuclear localization signal (NLS) that accounts for its directed location to the nucleus (62). Its roles in the nucleus have not been fully unraveled, but several functions have already been found, such as activating p53 (63) and limiting NF-κB signaling (64). Zimmer et al. adopted an elegant tool to study the nucleus-located SOCS1 by replacing its NLS. They found that mice lacking nuclear SOCS1, but not cytoplasmic SOCS1, do not have neonatal lethal symptoms, but have mild airway inflammation, indicating different roles of nucleus located and cytoplasm located SOCS1 (65).

SOCS1 is widely expressed in hematopoietic and stromal cells and therefore has multi-facet roles in immune regulation. In DCs, SOCS1 prevents aberrant overexpression of BAFF by breaking IFN induced “DC activation-IFNs release” positive loop, explaining autoimmune phenotypes, including overexpression of autoantibodies in SOCS1 deficient mice (66). In T cells, which are an important source of IFN-γ themselves, SOCS1 modulates their differentiation and terminates IFN-JAK-STAT signals to prevent overproduction of inflammatory cytokines (67, 68). SOCS1 is also found to sustain Foxp3 stability and Treg’s suppression function by preventing transformation to Th1- and Th17-like cells under inflammatory circumstances (69, 70). Deficiency of SOCS1 renders NKT cells abnormally active caused by loss of cross-talk inhibition of IFN-γ and IL-4 signaling, contributing to fulminant hepatitis (71).

Studies have shown a correlation between less SOCS1 serum levels and SLE. Abnormal activation of STAT1 in SLE patients contributes to over production of pro-inflammatory factors (72). Patients with heterozygous mutations in SOCS1 are reported to have immune cytopenia, autoimmune diseases, multisystem inflammation and lymphoproliferation (15, 16). Corresponding with the known function of SOCS1, it is observed that in patients with SOCS1 haploinsufficiency, phosphorylation of STAT1 after IFN-β and IFN-γ stimulation and basal expression of IFN-stimulated genes are increased compared to normal people, suggesting stronger type I and type II IFN signaling, which contributes to autoinflammation and cytopenia (15). Some SOCS1 mutation carriers are clinically asymptomatic, but still have an abnormal immune cell compartment, autoantibodies and higher IFN-γ-induced STAT1 phosphorylation (16).

A SOCS1 mimetic peptide alleviates SLE symptoms in MRL/lpr mice and significantly corrects their immune system by enhancing Foxp3 expression in Tregs and reducing abnormal T and B cell effects (73). Such peptides may also have therapeutic potential in SOCS1 deficient patients. Mycophenolate mofetil and the JAK1/2 inhibitor, baricitinib, are effective to mitigate manifestations in SOCS1 deficient patients (15, 16). Since SOCS1 expresses in both stromal and hematopoietic cells, the efficiency of HSCT remains unclear (15).

## TGFB1 Deficiency

Transforming growth factor (TGF)-β1 is encoded by *TGFB1* and is first translated into a precursor form containing an N-terminal signal peptide, a latency-associated peptide (LAP) and the C-terminal mature growth factor (TGF-β1). It is a strong immunosuppressive factor and functions through SMAD pathways (74). It has multifaceted roles in inflammation, oncogenic and fibrinogenic modulation. As for inflammatory regulation, TGF-β1 carried by extracellular vesicle mitigates inflammation in whole-blood cells by inhibiting *IL1B* transcription *via* upregulating SMAD7, as well as by amplifying the anti-inflammation role in endothelial cells *via* further upregulating *TGFB1* transcription (75). Although TGF-β1 is expressed by many types of cells, Tregs are a non-redundant source of it, controlling allergic and autoimmune responses in a microbiota- and dose-dependent way in mice. Dose-dependent refers to different phenotypes between *TGFB1* haploinsufficiency and biallelic deletion. *TGFB1* haploinsufficiency in Tregs leads to food allergies while biallelic *TGFB1* deletion results in autoimmunity, consisting of autoantibody release and dysregulations in DCs and effector T cells (76). Apart from Tregs, TGF-β1 also affects other subsets of T cells. It imposes constraints on activation-induced cell death (AICD) by downregulating Fas ligand *via* inhibiting c-Myc expressing, which is beneficial to the expansion of effector T cells and differentiation into memory T cells (77). It has a negative role in Th2 cell expansion, together with downregulating GATA-3 expression and IL-4-induced STAT6 activation (78). In cell populations other than T cells, TGF-β1 plays key roles as well. M2 macrophages not only bind to but also re-release TGF-β1, which is pivotal to their Treg induction role (79). In addition, TGF-β1 favors Langerhans cell (LC) differentiation in dendritic cells (DCs), while it shows a negative role in DC maturation, preventing DC activation in response to harmless environmental stimulation and therefore preparing them for response to dangerous signals (80). TGF-β inhibits maturation and activation of NK cells as well. It inhibits maturation from two facets, by preventing cell-cycle and by constraining transcription factors related to maturation (81). This inhibition has a double-sided nature. On one hand, it is useful for preventing harmful inflammation in early stages of development. And on the other hand, susceptibility to infection is extended since there is a lack of NK cells (81). A significant mediator of NK activation is mTOR induced by IL-2 or IL-15. By antagonizing the mTOR pathway, TGF-β negatively regulates activation of NK cells (82).

Typical manifestations of *TGFB1* deficient patients include inflammatory bowel disease (IBD) and recurrent infections which can be lethal are reported as well (17). Numbers and distributions for T cells, B cells and NK cells can be normal or disturbed while proliferation of T cells stimulated with anti-CD3 is reduced. The immune dysregulation phenotypes of TGF-β1 deficient patients suggest an indispensable role of TGF-β1 in immune regulation. Besides, central nervous system (CNS) dysfunctions are also common in *TGFB1* deficient patients. These dysfunctions include epilepsy, brain atrophy and posterior leukoencephalopathy (17). Former studies show reduced neuronal TGF-β signaling promotes Alzheimer’s disease (AD) and neurodegeneration (83). Mechanisms underlying neuronal roles of TGF-β has not been totally revealed. One possible explanation lies in its relationship with olfactory ensheathing cells (OECs). Clearance of degenerating or dying neurons and apoptotic neuron debris is critical to neuron regeneration, that is to say, it benefits the restoration of CNS injuries and neurodegeneration. TGF-β promotes this process by enhancing phagocytic activity of OECs through integrin/MFG-E8 signaling pathway and by shifting OECs shape to increase cellular surface area (84).

Possible therapies include HSCT and recombinant TGF-β1 replacement, both of which have risks of severe comorbidities, and thus there is a need for safer and more efficient treatments (17). As for the symptoms of early onset IBD, surgery, nutritional therapy and some emerging new methods like complementary medicine and fecal microbiota transplantation may also be effective (85).

## RIPK1 Deficiency

RIPK1 (receptor-interacting serine/threonine kinase 1) is a component of signal transduction complexes, mediating signals from surface receptors like Toll-like receptor 3 (TLR3), TLR4 and tumor necrosis factor receptor 1 (TNFR1) and controlling cell death and inflammation. RIPK1 can both mediate cell death and promote cell survival (86). Its role in cell death varies with cell types and contexts. There are two types of cell death inhibited by RIPK1 in hematopoietic cells, keratinocytes, epithelial cell and DCs, namely necroptosis *via* RIPK3 and pseudo-kinase mixed lineage kinase domain-like (MLKL), and apoptosis *via* caspase-8. These two types can switch from one to the other easily, when responding to conditional changes (86–88). Because necroptosis results in proinflammatory danger-associated molecular patterns (DAMP), RIPK1 can act as an inhibitor of inflammation in physiological situations (89–91). RIP homotypic interaction motif (RHIM) of RIPK1 is indispensable in competing with RIPK3 RHIM in ZBP1 (Z-DNA binding protein 1; also known as DAI or DLM1) binding and thus prevents RIPK3 autophosphorylation that is critical for necroptosis (92, 93). In the regulation of caspase-8, RIPK1 acts as both scaffold and kinase (88, 94, 95).

Manifestations in human patients include lymphopenia, susceptibility to infections, early-onset IBD and arthritis. Numbers of T, B and NK cells diminish to different extents (18–20). Cellular and molecular studies showed that patients with loss-of-function mutations in *RIPK1* have dysregulation in cytokine release such as increased IL-1β and decreased IL-10, contributing to arthritis and IBD together (19). Although *RIPK1* conditional deletion animal models show autoimmunity, patients with RIPK1 deficiency have not been reported to have autoimmune symptoms (90, 91).

A consensus has not been reached in therapeutic approaches for RIPK1 deficiency. Cuchet-Lourenço et al. proposed that HSCT is adequate since it greatly remitted clinical symptoms and dysregulated cytokine production in a patient (19), while Li et al. warranted that intrinsic intestinal phenotypes cannot be mitigated by HSCT (20).

In recent years, RIPK1 inhibitors are considered to be a curative therapy to diseases related to necroptosis. Studies with RIPK1 deficient patients further reveal the multi-faceted roles of RIPK1, so the use of RIPK1 inhibitors should be cautious and its safety requires deeper research (20). Besides, the lack of consensus on RIPK1 deficiency treatment also emphasizes the urgency of revealing the exact functions of RIPK1 in human.

## CD137 Deficiency

CD137, also known as 4-1BB or tumor necrosis factor (TNF) receptor superfamily member 9 (TNFRSF9) is pivotal for immune homeostasis and tumor suppression. Ligation by CD137L leads to oligomerization of CD137 and galectin-9 (Gal-9) serves as a bridge. TRAFs are then recruited and activate NF-κB and PI3K-AKT pathways (96). Although it is expressed by various types of cells, its role in T cells as an inducible costimulator has been studied the most, which is significant for proper T cell survival, differentiation and cytokine secretion (97). The pro-survival role is mediated by upregulated anti-apoptosis protein Bcl-x_L_ through the NF-κB pathway (97, 98). Generally, CD137 positively regulates the T cell response, but there are some exceptions. In the case of Tregs, it promotes clonal expansion, yet transiently neutralizes the suppressive activity of activated Tregs. Also, CD137 inhibits the expansion of Th17 in both IFN-γ dependent and independent ways, which is important for remission of autoimmunity (99). CD137 is also expressed on human B cells, but not murine B cells. Its functional mode in B cells resembles that in T cells in several facets. First, activation of BCRs/TCRs is a prerequisite for CD137 expression, which enhances survival, proliferation and cytokine production (97, 100). In invariant NKT (iNKT) cells, iNKT–monocyte interaction *via* CD137/CD137L promotes iNKT survival and proliferation and this interaction also affects monocytes survival (101). When CD137 is deficient, iNKT counts are reduced (102) due to at least partially the attenuated CD137/CD137L signal. Since iNKTs provide apoptosis signal to monocytes and prevent their over proliferation during inflammation, reduced iNKT number may account for the immune dysregulation of CD137 deficient patients. Additionally, like many TNF/TNFR superfamily members, CD137/CD137L evokes reverse signals to CD137L positive cells, making it hard to determine the respective functions of each one (103).

CD137 deficient mice have enhanced T cell proliferation, while the CTL response and IFN-γ expression are dampened (104). Patients having reduced or ablated expression of CD137 show hampered immune regulation, being susceptible to various pathogens including Epstein-Barr virus (EBV) and predisposed to EBV-related B-cell lymphoma as well as showing symptoms of autoimmunity (21, 22). Proliferation and function of both B cells and T cells are dysregulated, emphasizing the importance of CD137 in homeostasis of immune system. Notably, mitochondrial function is significant in T cell function and it is impaired in CD137 deficient patients (22). However, it is intriguing that some siblings harboring the same mutation as the patients do not show clinical manifestations, suggesting that the overt disease is affected by some other factors other than genes (21).

Possible therapeutic approaches lie in other costimulators like CD28, given that elevated CD28 signals ameliorated deficiency in T cell proliferation (21). Furthermore, the immunophenotype can be reversed by HSCT. As for patients with lymphoma, the less toxic and more specific therapies are recommended before HSCT, which can prevent the use of radiological treatment (23).

## TET2 Deficiency

Ten-Eleven Translocation methylcytosine dioxygenase 2 (TET2) is a pivotal epigenetic regulatory factor in hematopoietic cells and it facilitates demethylation by oxidizing 5-methylcytosine (5mC) to 5-hydroxymethylcytosine (5hmC) and other oxidation products (24). Accumulating evidence points out that its mutations are frequent in clonal hematopoiesis and myeloid malignancies (25). Therefore, it is reasonable to presume that TET2 has a crucial role in cell proliferation and differentiation. Homogeneous or heterogeneous loss of TET2 renders stem cell renewal more active in a cell intrinsic way, disturbs differentiation and increases the risk of myeloproliferation (105–108). CXCR4, a chemokine receptor critical for B cell development, is elevated in heterozygous TET2 mutant patients, suggesting a B cell regulatory role of TET2 (109). TET proteins are also essential for specific points of B cell development, for example the transition from pro-B to pre-B and the differentiation to plasma cells. For the former one, TET2/3 not only augment *Igκ* expression and rearrangement per se, but also assist the function of transcription factors (110). By demethylating CpG sites around *Irf4*, they allow high expression of IRF4, which is critical for the transition into plasma cells. However, it is not necessary for the initiation of *Irf4* expression, but only the maintenance of it (111). In Tregs, TET is crucial for stable Foxp3 expression through regulation on the conserved non-coding DNA sequence-2 (CNS2) region and ‘upstream enhancer’ region. TET2/3 double-knockout Tregs show abnormal activity in proliferation and the counts of Th17 and Tfh-like cells are increased as well (112).

Autosomal homozygous TET2 missense or nonsense in humans results in immunodeficiency, growth impairment and autoimmune lymphoproliferative syndrome (ALPS) like phenotypes of raised proportion of double negative (CD4^-^CD8^-^) T-cells (DNTs), raised soluble Fas ligand level, lymphadenopathy, hepatosplenomegaly, autoimmunity and remarkable predisposition to lymphoma, which is reminiscent of phenotypes of TET2 deficient mice (24). Due to the loss-of-function mutation of TET2, levels of DNA methylation increase in hematologic cells, especially in the regions able to bind master transcription factors that have a strong regulatory effect on hematopoiesis, accounting for the skew to DNTs and failure of proper development in B cells (24, 109). Increased soluble Fas ligand accounts for reduced FasL-induced apoptosis and therefore the tendency to lymphoproliferation. Haploinsufficiency of TET2 is a wide spread mutation related to hematological neoplasia, although it is not able to induce cancer alone given that *TET2* mutations also occur in healthy groups with clonal hematopoiesis (25, 109, 113, 114). Extrinsic factors may explain the phenotypes, but the underlying mechanisms have not been fully unraveled. Recent studies show that infection-induced inflammation is critical for the onset of malignance in TET2^-/-^ mice, shedding light on the clinical prevention and treatment of malignancy related to TET2 deficiency (115, 116). In diffuse large B cell lymphomas (DLBCLs), gene alterations involved in TET2 mutation consist of both losing enhancer 5hmC and gaining promoter 5mC. Additionally, the chromatin accessibility and stability are also reduced. Activity of activation-induced cytidine deaminase (AID) and subsequent deamination are hampered in *TET2^-/-^* mice, which contributes to the disturbance of demethylation further. Together, these changes disrupt transcription of genes critical for GC (germinal center) exit, antigen presentation and differentiation of GC B cells, accounting for the occurrence of DLBCLs (113). In addition, mutations in TET2 are also a risk factor for neurodegenerative disorders such as early-onset Alzheimer’s disease and frontotemporal dementia (117).

Recent studies have shown that vitamin C can mimic restoration of TET2, implicating a possible effect of high-dose vitamin C incorporation on TET2 deficient patients. Notably, the function of vitamin C requires a minimal existence of TET, meaning that the combined loss of TET2 and TET3 leads to poor response to vitamin C treatment (107). Besides, since mutations in TET2 alone are not sufficient for cancer onset, therapy targeting assistant factors, such as other genetic deficiencies and immunostimulation, are also effective treatments. As mentioned before, infection-induced inflammation, which can be corrected by antibiotics, shows positive correlation with myeloid expansion in TET2 deficient mice. Inhibition of bacterial inflammatory signals, such as inhibiting TNF-α, prevents tumor growth, providing a viable preventive method for TET2 deficient malignancy (116). With the high level of methylation being a positive factor for malignancy, it is not surprising that DNA methyltransferase inhibitors (DNMTi) are also effective (113).

## Conclusion

In recent years, there has been a rapid growth of IEI discoveries and a deeper understanding of their mechanisms. It has come to our knowledge that IEI does not merely mean higher susceptibility to infections. Those patients with a combination of several types of autoimmune or inflammatory diseases should be considered for a scan of IEI. As for therapeutic methods, nonspecific immunosuppressive agents used to prevent autoimmunity sometimes have many side effects and they do not fit to the situation where autoimmune and susceptibility coexist. HSCT has similar shortcomings as well. Morbidity and mortality related to HSCT are still considerable problems (118). Besides, although it has a definitive effect in some cases, it acts less well when stromal cells, but not merely blood cells, are also affected (15). Thus, safer and more effective therapeutic methods are required. Pathogenesis studies of these immune dysregulations will shed light on this field. Clarifying the relationship between IEI and immune dysregulation helps not only to diagnose patients as early as possible, but also to improve the quality of the patients’ life and prolong their survival. Apart from the direct clinical use, studies on IEI also provide a wonderful way to understand the mechanism of immune regulation in our body and produce animal models for related research (10, 33).

There are several difficulties in identifying IEI: First, since every single inborn error has a relatively low prevalence rate, it is hard to figure out its clinical symptoms fully and quickly. Some patients harboring more than one mutation make it even more difficult to detect (11). Besides, it is intriguing and confusing that phenotypes of the same mutation vary from patient to patient, ranging from mild or even no obvious symptoms to life-threatening manifestations (21, 119). This complexity may come from both internal and external factors, such as incomplete penetrance and additional factors like infections and combination with other gene deficiencies (20, 21, 120).

The most crucial issue in IEI is to identify their pathogenesis and gene-phenotype relationship. It requires both clinical and laboratory efforts to address this problem. In clinical treatment, patients with immune dysregulations should be examined carefully and genetic identification should be executed if necessary and possible. By doing so, it can not only avoid misdiagnosis, but also find new mutations. Considering that the prevalence of IEI is very low and acquiring enough samples for studies is difficult, creating proper animal models is a useful alternative. However, it should be kept in mind that there are differences between humans and animals. Therefore, results from animal models only provide us with an indication and they should be examined on humans later to test the consistency.

## Author Contributions

AR wrote the article. WY, HM, LW, FC, CP and PL reviewed and revised the draft. QG, YC and CL organized and revised the draft. All authors contributed to the article and approved the submitted version.

## Funding

This work was supported by National Natural Science Foundation of China (31970839), HUST Academic Frontier Youth Team (2018QYTD10) and Independent Innovation Research Fund of Huazhong University of Science and Technology (2020kfyXGYJ017).

## Conflict of Interest

The authors declare that the research was conducted in the absence of any commercial or financial relationships that could be construed as a potential conflict of interest.

## Publisher’s Note

All claims expressed in this article are solely those of the authors and do not necessarily represent those of their affiliated organizations, or those of the publisher, the editors and the reviewers. Any product that may be evaluated in this article, or claim that may be made by its manufacturer, is not guaranteed or endorsed by the publisher.
